# Glioma-associated mesenchymal stem cells-mediated PD-L1 expression is attenuated by Ad5-Ki67/IL-15 in GBM treatment

**DOI:** 10.1186/s13287-022-02968-z

**Published:** 2022-06-28

**Authors:** Qing Zhang, Junwen Zhang, Peiwen Wang, Guidong Zhu, Guishan Jin, Fusheng Liu

**Affiliations:** 1 Brain Tumor Research Center, Beijing Neurosurgical Institute, Capital Medical University, Beijing, 100070 People’s Republic of China; 2 Department of Neurosurgery, Beijing Tiantan Hospital Affiliated to Capital Medical University, Beijing, 100070 People’s Republic of China; 3 Beijing Laboratory of Biomedical Materials, Beijing, 100070 People’s Republic of China

**Keywords:** Glioma-associated mesenchymal stem cells, PD-L1, Glioblastoma, Oncolytic virus, Angiogenesis

## Abstract

**Background:**

Glioblastoma (GBM) is a highly immunosuppressive and vascular malignant brain tumor. Current therapeutic strategies targeting tumor cells have limited efficacy because of the immunosuppressive microenvironment and vascularization. Glioma-associated mesenchymal stem cells (GA-MSCs) have been identified as important stromal components of the tumor microenvironment, owing to their contribution to tumor angiogenesis and their potential to drive glioma stem cells. However, there are no reports on the effect of oncolytic Ad5-Ki67/IL-15 on programmed death ligand 1 (PD-L1) expression and angiogenesis induced by GA-MSCs.

**Methods:**

Flow cytometry was respectively performed to detect the PD-L1 of glioma cells and programmed death protein 1 (PD-1), CD3, CD4 and CD8 in lymphocytes, as well as distribution of the cell cycle. CCK-8 assay investigated the proliferation of glioma cells and GA-MSCs in vitro. Tumor-bearing nude mice were established with U87-Luc cells and treated with the viruses, and further the IVIS spectrum was utilized to obtain luciferase images. Finally, the expression of PD-L1 in tumor tissues was also investigated using western blotting.

**Results:**

We found that GA-MSCs had potential to induce PD-L1 upregulation and involved in vascular mimicry in vitro. Importantly, Ad5-Ki67/IL-15 reduced PD-L1 expression of glioma cells and neovascularization by targeting GA-MSCs. Furthermore, despite the presence of GA-MSCs, the virus has the ability to generate potent antitumor efficacy in vitro and vivo.

**Conclusions:**

These findings suggest the use of oncolytic Ad5-Ki67/IL-15 targeting GA-MSCs to treat GBM, indicating potential clinical applications.

**Supplementary Information:**

The online version contains supplementary material available at 10.1186/s13287-022-02968-z.

## Highlights

1. Glioma-associated mesenchymal stem cells are important stromal components, which involve in PD-L1 upregulation in gliomas.
2. The oncolytic virus Ad5-Ki67/IL-15 has the potential to attenuate GA-MSCs-mediated PD-L1 expression and angiogenesis.
3. In animal model, the Ad5-Ki67/IL-15 can overcome GA-MSCs to generate antitumor efficacy.
4. In personalized medicine, further thorough work will provide a potential new approach targeting stroma components in the treatment of patients with glioma.

## Background

Glioblastoma (GBM) is the most aggressive and lethal brain tumor, and patients with GBM have extremely poor prognosis and an expectancy time of almost 14.6 months [1, 2]. Current treatments for glioma include radiation and chemotherapy, for instance, temozolomide and bevacizumab and aim to kill tumor cells and inhibit tumor neovascularization [3–6]. However, there have been few studies of treatment strategies targeting surrounding stromal cells such as mesenchymal stem cells (MSCs). Glioma-associated MSCs (GA-MSCs) have been identified as stromal components that contribute to tumorigenesis and have the potential to drive glioma stem cells (GSCs), particularly in the unique microenvironment of human brain tumors [7, 8]. GA-MSCs are important mesenchymal cells in glioma microenvironment. Furthermore, the fraction of GA-MSCs in human high-grade gliomas is inversely correlated with the overall survival time of patients [9]. In addition, the function of GA-MSCs in glioma progression is associated with CD90 expression [10]. However, the immunomodulation roles of GA-MSCs remain to be elucidated.

Recent studies reveal that bone marrow mesenchymal stem cells (BM-MSCs) have crucial roles in determining immunosuppressive characteristics [11–14]. BM-MSCs have been reported to secrete immunosuppressive factors including prostaglandin E2 (PGE2), transforming growth factor beta (TGFβ), and interleukin-10 (IL-10), as well as intracellular enzyme indoleamine 2,3-dioxygenase (IDO), resulting in suppression of proliferation, activation and differentiation of T cells [15]. In addition, these mesenchymal stem cells (MSCs) produce various chemokines and express adhesion molecules that are responsible for immune cell recruitment and maintaining close proximity with immune cells [16, 17]. Furthermore, the suppressive effect of MSCs on T cells is thought to be mediated by the release of TGFβ and hepatocyte growth factor (HGF), leading to a reduction in cyclin D2 and an increase in p27kip1 levels in T cells, with consequent arrest of the cell cycle in the G1 phase [18, 19]. Nevertheless, the roles of GA-MSCs in glioma treatment have not well expounded. Therefore, deeper understanding of the immunological roles of GA-MSCs will facilitate the development of new strategies for the treatment of gliomas.

Programmed death ligand 1 (PD-L1), which is present in the tumor microenvironment (TME), is an immune inhibitory receptor ligand that leads to immune cell dysfunction and apoptosis by binding to its receptor, programmed death protein 1 (PD-1), which works in braking the inflammatory response and conspiring tumor immune evasion [20, 21]. PD-1, another immune checkpoint receptor, is expressed on activated immune cells, which usually upregulated in the TME [22, 23]. In addition, GBM is a malignant brain disease that abundant with vascular structure. Tumor angiogenesis is one of the important factors that lead to rapid growth and progression of glioma. Thus, it is critical to develop strategies that will enhance immune response during treatment and expand the range of brain gliomas that can be effectively treated.

Oncolytic virus (OV) therapy is a promising therapeutic approach for solid tumors that involves selectively infecting and killing tumor cells [24, 25]. Most previous studies of OVs have focused on tumor cells, but the research about OVs targeting GA-MSCs have not been elucidated. Therefore, we designed a novel oncolytic adenovirus Ad5-Ki67/IL-15 to explore its potential therapeutic applications for targeting MSCs. Our previous work demonstrated that Ad5-Ki67/IL-15 selectively killed tumor cells and exhibited potent antiangiogenic capacity via reduction of VEGF secretion [25].

Here, we reveal that GA-MSCs contribute to PD-L1 upregulation in GBM. Furthermore, Ad5-Ki67/IL-15 can reduce GA-MSC-mediated PD-L1 expression and angiogenesis in glioma. In GBM model, the virus can effectively inhibit tumor growth even though the presence of GA-MSCs. This research is innovative because the immunosuppressive effects of GA-MSCs have not previously been explored or tested. These results indicate a potential new approach for the treatment of glioma, using Ad5-Ki67/IL-15 to target not only tumor cells but also GA-MSCs.

## Materials and methods

### Isolation and culture of GA-MSCs

Isolation of human GA-MSCs was performed as described previously [10]. Briefly, the GA-MSCs were separated from the fresh glioma specimens; the tissue was cut into pieces after washed twice in PBS. Afterward, the trypsin was added to the pieces for digestion. Then, the cell suspension was transferred to a 70-μm filter and washed twice again with PBS. GA-MSCs were cultured in DMEM containing 10% fetal bovine serum (FBS; Gibco, USA) and 100 U/ml penicillin/streptomycin (Gibco, USA) in a humidified atmosphere at 37 °C with 5% CO_2_. All experiments were performed according to the flow chart (Additional file 1).

### Mouse lymphocytes

Isolation of mouse spleen lymphocytes was performed according to the instructions. Briefly, fresh spleen was obtained and transferred to a 70-μm filter (Corning, USA) on a 50-ml centrifuge tube, then gently ground, washed continuously with precooled PBS and centrifuged at 1800 rpm for 5 min. The cell pellets were re-suspended in washing solution and separated with lymphocyte fluid at a 1:1 ratio density gradient centrifugation at 2000 rpm for 20 min. The collected cells were washed using washing buffer and cleaning buffer before use in experiments.

### Construction of Ad5-Ki67/IL-15

The recombinant oncolytic adenovirus (OAd) has been previously described [25]. Briefly, an intrinsic promoter that controls the type 5 adenovirus E1A gene was replaced by the Ki67 promoter sequence, and GFP gene was inserted into E3 region, thus forming Ad5-Ki67/GFP. Subsequently, GFP gene was replaced with human IL-15 gene to produce Ad5-Ki67/IL-15. Therefore, the targeted OAd Ad5-Ki67/IL-15 was constructed.

### Glioma cells

GL261, U87, U251 and BT-01 were cultured in DMEM (Gibco, USA) containing 10% FBS (Invitrogen, China) and 100 U/ml penicillin/streptomycin (Gibco, USA) in a humidified atmosphere at 37 °C with 5% CO_2_.

### Collection of conditioned media

GA-MSCs, U251 and BT-01 cells were seeded in six-well plates containing the viruses at a multiplicity of infection (MOI) of 40 for 72 h. The supernatants were collected and centrifuged at 2000 rpm for 10 min to remove cells and cellular debris.

### Immunofluorescence

GA-MSCs were fixed with 4% paraformaldehyde, permeabilized with 0.5% Triton, and blocked with 3% bovine serum albumin for 1 h at room temperature. The cells were incubated with primary antibodies against CD44 and CD105 (1:100, Proteintech, China), then overnight at 4 °C. The secondary antibodies used were as follows: Cy3-conjugated antibodies (1:100, Boster, WuHan, China). Nuclei were stained for visualization using DAPI. Immunofluorescence microscopy was performed with an Olympus microscope.

### Fluorescence microscopy

GA-MSCs were treated with Ad5-Ki67/GFP at an MOI of 40 and were observed under an Olympus microscope. Images were taken 48 h after infection.

### Cell cycle

The GA-MSCs (1 × 10^5^ cells/well) were seeded into 6-well plate with 10% FBS (Invitrogen, China) and 100 U/ml penicillin/streptomycin (Gibco, USA) in a humidified atmosphere at 37 °C with 5% CO_2_. After the cell adherence, the culture condition was added with the virus Ad5-Ki67/IL-15 (MOI = 40), and incubated for 72 h. The untreated cell was as a control. The cells were fixed in 95% ethanol (4 °C) overnight. Then, the fixed cells were washed twice with precooled PBS and incubated in 2.5 mg/ml DNase-free RNase A (Gene-Protein Link) and PI (1×, Gene-Protein Link) for 30 min at 37 °C. The distribution of the cell cycle was detected using a flow cytometry.

### Cell viability

Cell viability was analyzed using the Cell Counting Kit-8 (CCK-8 Kit, Dojindo Laboratories, Japan)**.**

The cells (3000 cells/well) were seeded into a 96-well plate and cultured overnight. Then, the medium was replaced with 100 µl of different media and cultured for 1, 2, 3 days. At every time point, 10 µl of CCK-8 was added to each well and incubated for 2 h at 37 °C with 5% CO_2_. Then, the absorbance of each well was measured at 450 nm using a microplate reader (PerkinElmer, USA). At least three wells were used for each sample in different media.

### Tube formation assay

Tube formation assays were performed according to previous descriptions [25]. Briefly, growth-factor-reduced Matrigel (BD, USA) was pre-added to 96-well plates. Cells (2 × 10^4^ cells/well) were seeded into wells and incubated at 37 °C and 5% CO_2_. After 6 h, the cells were labeled using Calcein AM (Tocris, USA), and tube formation was imaged with an Olympus microscope. Capillary-like tube formation of GA-MSCs was analyzed in three random fields of view per well using ImageJ software (NIH, USA). The tube segment lengths and number of tubes of GA-MSCs cultured with different conditions were also quantified.

### Coculture assay

For the coculture experiments, glioma cells GL261, U251, U87 and BT-01 (1 × 10^5^ cells/well) and lymphocytes (1 × 10^6^ cells/well) were, respectively, placed into the bottom wells of transwell permeable 6-well plate supports (Corning, Corning, NY), then the GA-MSCs (1 × 10^5^ cells/well) were plated in the apical chamber, and the virus Ad5-Ki67/IL-15 (4 × 10^6^ VP) was added into the upper chamber, which was co-cultured for 72 h. Inserts had a pore size of 0.4 μm, permitting the free exchange of molecules but preventing cell migration or contact. The experiments included different groups: cultures of glioma cells with addition of GA-MSCs, cultures of glioma cells with GA-MSCs plus virus, glioma cells with single virus and only glioma cells. All cells were cultured in a supplemented MSCs basal medium. After 3 days in coculture, the cells were collected to perform flow cytometry analysis.

### Flow cytometry

Flow cytometry analysis was performed using fluorochrome-conjugated antibodies. Cultured glioma cells were detached with 0.05% trypsin–EDTA (ATCC), and enzymatic action was stopped by adding 10% FBS in PBS (Gibco, USA). The cells were washed in PBS, and then, the pellets were re-suspended in fluorescent-activated cell sorting (FACS) buffer. The glioma cell suspensions were stained with APC-conjugated antibodies against human or mouse PD-L1 (1:20, Proteintech, USA), and the lymphocytes were, respectively, stained with mouse PD-1 (1:100, Proteintech, USA), CD3, CD4 and CD8 (1:100, BioLegend, USA). All cells were incubated in the dark at 4 °C for 20 min. Then, the cells were centrifuged and re-suspended in PBS. Data were acquired within 2 h after staining on a BD Accuri C6 Plus (BD Biosciences), and analyzed using FlowJo software (Tree Star, Ashland, OR, USA).

### Animal experiments

All animal studies were approved by Experimental Animal Welfare and Ethics Committee of Beijing Tiantan Hospital affiliated with Capital Medical University and approved all the animal experiments. Male BALB/nu-c (6 weeks old; Beijing Vital River Laboratory Animal) were kept in the animal facilities at Beijing Neurosurgical Institute and maintained under specific-pathogen-free conditions. Inhalation of isoflurane was used to anesthetize the animals in all experiments. BALB/nu-c (*n* = 5/group) was subcutaneously inoculated with a PBS suspension containing 1 × 10^6^ U87-Luc cells (100 ul) in the right underarm using 1 ml syringe. The GA-MSCs suspension (100 ul, 1 × 10^5^ cells) or Ad5-Ki67/IL-15 (3 × 10^11^ vp) plus GA-MSCs (1 × 10^5^ cells) were injected into the tumor sites 14 days after U87-Luc cell implantation. Tumor volumes were calculated according to the following formula: width^2^ × length × 0.5.

### IVIS imaging

The IVIS spectrum was utilized to obtain luciferase images. In vivo images were obtained on day 0 (prior to virus injection) and on days 3 and 6 after virus and GA-MSCs injection. Each mouse was treated with 100 μl 15 mg/ml d-luciferin (PerkinElmer, Waltham, MA, USA) i.p. in PBS.

### Western blotting

The animals were anesthetized and sacrificed after treatment for 10 days. The tumor specimens were harvested from the mice, then washed two times and cut into pieces. The total protein was extracted with RIPA lysis buffers were treated by ultrasound and supplemented with mammalian protease inhibitor (Sigma-Aldrich, St Louis, MO, USA). Protein samples (20 μg per lane) were loaded into gels for separation via sodium dodecyl sulfate polyacrylamide gel electrophoresis. After separation, the proteins were transferred to nitrocellulose membranes (Thermo, Waltham, MA, USA), which were then blocked for 60 min at room temperature in TBST (Tris-buffered saline, 0.1% Tween 20) containing 5% nonfat milk. Membranes were washed with TBST and probed with primary antibodies against PD-L1 (1:800, all from Santa Cruz Biotechnology, USA) at 4 °C overnight. Membranes were washed with TBST and incubated with secondary antibodies for 1 h at room temperature. Signals were detected with an ECL detection system.

### Statistical analysis

Statistical analyses were performed using GraphPad Prism 6.0. Sample sizes for each experiment are indicated in the Results or the Material and Methods section. The specific statistical tests used were t test for single comparisons and analysis of variance followed by Tukey’s test for multiple comparisons, and *P* < 0.05 was considered to indicate statistical significance. Numerical values are reported as mean ± standard deviation.

## Results

### GA-MSCs contributed to mediate PD-L1 expression in glioma cells

**Fig. 1:**
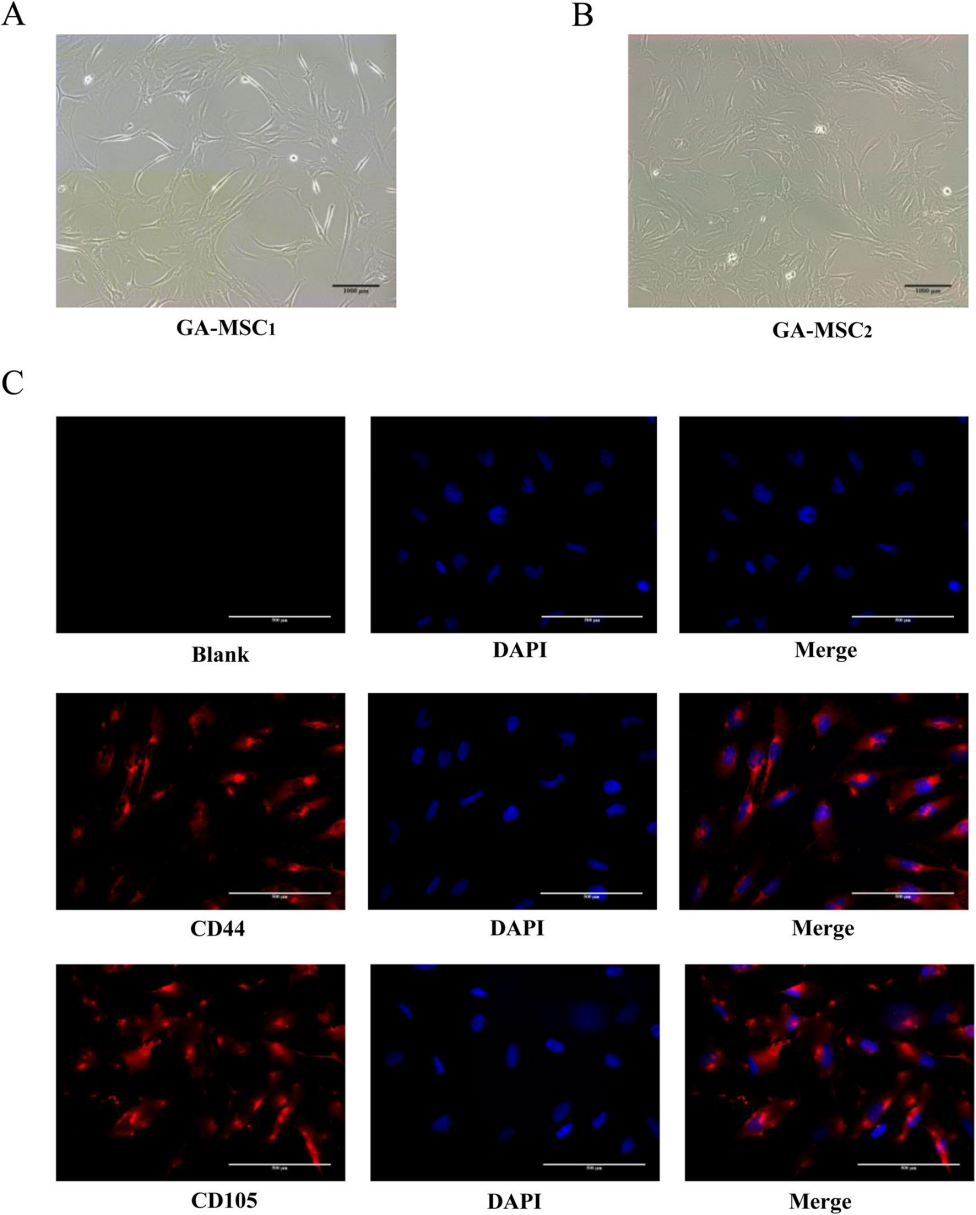
Identification of MSCs derived from human glioma tissues. **A** and **B** Adherent growth patterns and fibroblastic morphology of GA-MSCs cultured in MSC media (× 100, scale bars = 1000 µm). **C** Immunofluorescence showed that GA-MSCs expressed MSC specific markers CD44 and CD105 (× 400, scale bars = 500 µm)

**Fig. 2:**
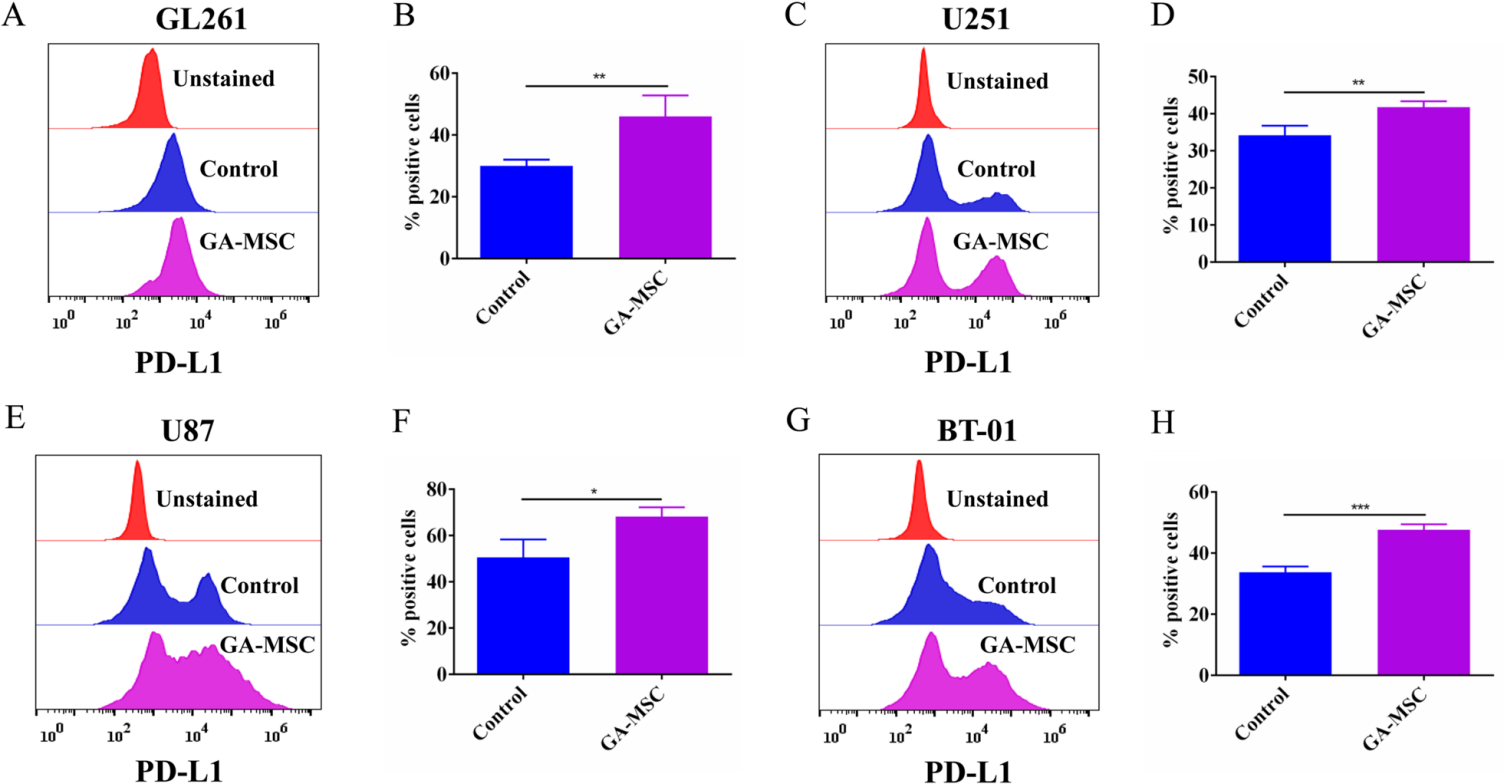
GA-MSCs had the ability to upregulate PD-L1 expression of glioma in vitro. **A** and **B** Mouse glioma cell GL261 cocultured with GA-MSCs for 72 h. Flow cytometry showed that PD-L1 expression was increased by GA-MSCs compared with control. **C** and **D** Human glioma cell U251. **E** and **F** Human glioma cell U87. G-H. Human glioma cell BT-01. All data are presented as mean ± SD of three independent experiments. *P < 0.05, **P < 0.01, ***P < 0.001

GA-MSCs were isolated from human fresh glioma specimens and showed similar classical MSC characteristics with fibroblastic morphology in standard medium in vitro (Fig. 1A, B). Immunofluorescence demonstrated that the GA-MSCs expressed MSC markers CD44 and CD105 (Fig. 1C). To explore GA-MSC-mediated PD-L1 expression of glioma, mouse glioma cell GL261 were co-cultured with GA-MSCs for 72 h. Flow cytometry showed that PD-L1 expression in GL261 cells was significantly upregulated compared with the control (Fig. 2A, B, *P* < 0.01, 30.03 ± 2.065 vs. 46.03 ± 6.793). Identically, human glioma cells U251 (*P* < 0.01, 34.2 ± 2.563 vs. 41.83 ± 1.582), U87 (*P* < 0.001, 50.6 ± 7.754 vs. 68.17 ± 4.055) and BT-01 (*P* < 0.001, 33.77 ± 1.882 vs. 47.67 ± 1.818) were, respectively, cocultured with GA-MSCs for 72 h. We also found that GA-MSCs contributed to mediate PD-L1 upregulation in human glioma cells (Fig. 2C–H).

### The effects of Ad5-Ki67/IL-15 on growth and angiogenesis of GA-MSCs

**Fig. 3:**
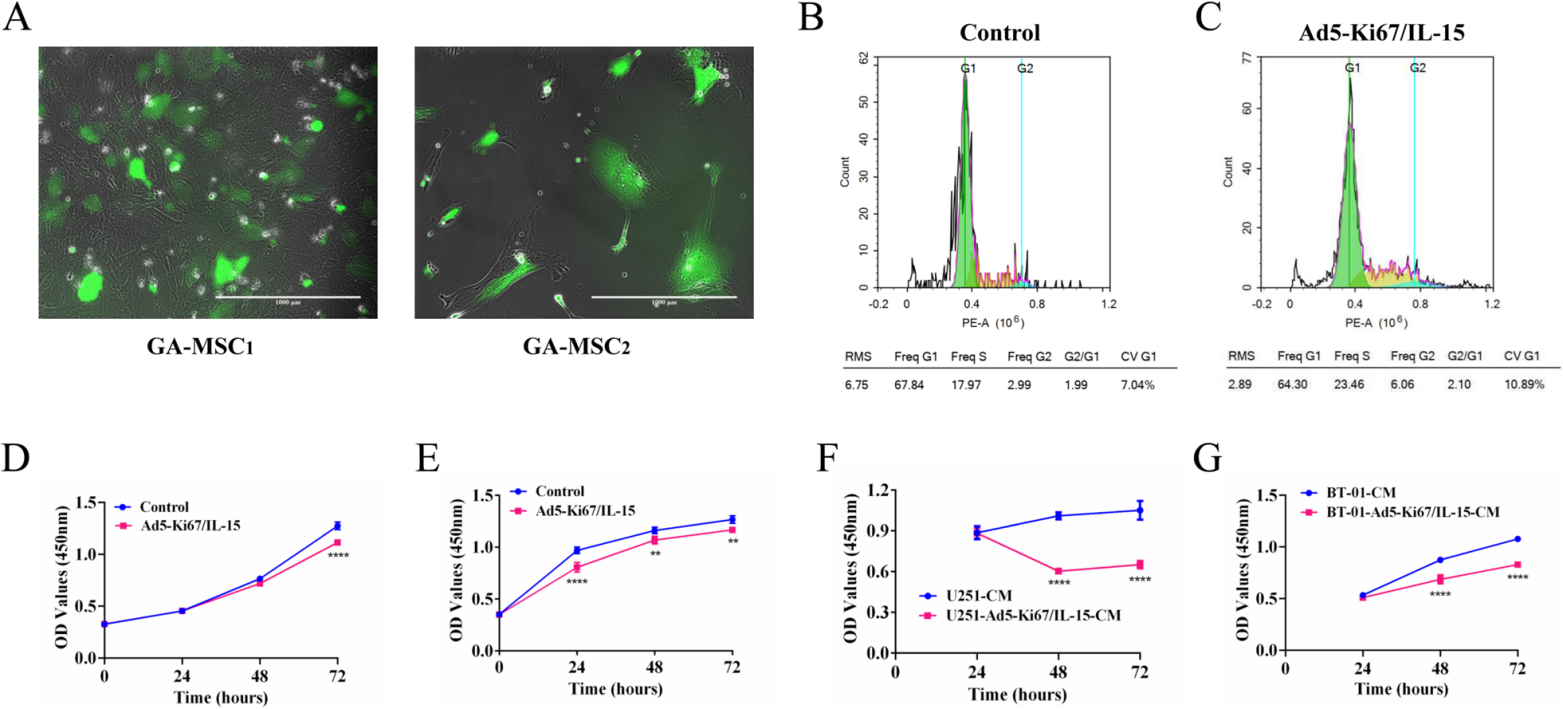
Ad5-Ki67/IL-15 effectively infected and killed GA-MSCs in vitro. **A** The GA-MSCs treated with the virus (MOI = 40) for 48 h, the representative images were acquired using fluorescence microscopy (× 100, scale bars = 1000 µm). **B** and **C** The cell cycle distribution of GA-MSCs treated with Ad5-Ki67/IL-15. **D** and **E** CCK-8 assay was performed to evaluate cell growth of GA-MSCs that treated with Ad5-Ki67/IL15 (MOI = 40) for 24, 48, 72 h. Ad5-Ki67/IL15 could inhibit the proliferation of GA-MSCs compared to control. **F**-**G** The proliferation of GA-MSCs in response to the conditioned medium from the virus-treated U251 or BT-01 cells (U251/BT-01-Ad5-Ki67/IL15-CM, MOI = 40) at different time points was determined by CCK8. The conditioned medium of glioma cell-treated with the virus significantly suppressed the cell growth compared with the untreated. All data are presented as mean ± SD of three independent experiments. *P < 0.05, **P < 0.01, ***P < 0.001, ****P < 0.0001

To address the effects of the virus on GA-MSCs, we performed infection and proliferation assays for the cells. We found that the Ad5-Ki67/GFP can infect GA-MSCs (Fig. 3A). Furthermore, the cells were cocultured with the virus Ad5-Ki67/IL-15 and detected the cell cycle distribution by flow cytometry. The proportion of cell arrest in S (23.46%) and G2 (6.06%) phase in Ad5-Ki67/IL-15 was increased compared to the control (S: 17.97%, G2: 2.99%) (Fig. 3B, C). CCK-8 assay displayed that Ad5-Ki67/IL-15 has the potential to suppress their growth (Fig. 3D, E). Furthermore, the conditioned media from glioma cells treated with Ad5-Ki67/IL-15 significantly inhibited proliferation of GA-MSCs (Fig. 3F, G).

**Fig. 4:**
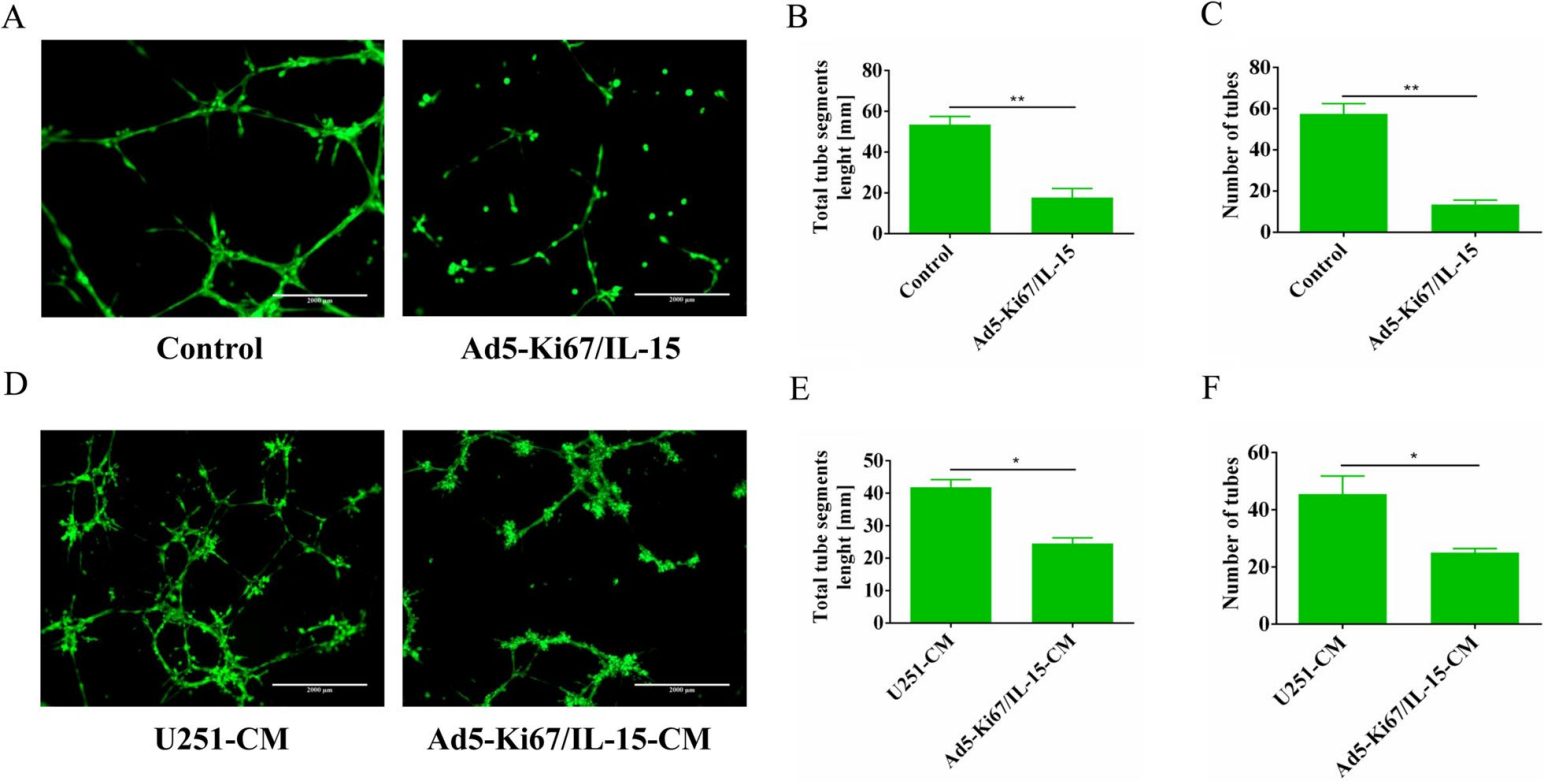
Ad5-Ki67/IL-15 reduced angiogenic capacity of MSCs. **A**–**C** Tube formation capacity of GA-MSCs cultured with viruses. The viruses inhibited the formation of vascular-like structures by GA-MSCs. **D**–**F** Angiogenic capacity of GA-MSCs incubated in conditioned media from GL261 treated with viruses. Conditioned media from U251 cells treated with viruses decreased GA-MSC angiogenesis (× 50, scale bars = 2000 µm). All MOI = 40. *P < 0.05, **P < 0.01

Tube formation assays performed in vitro showed that tube formation capacity was significantly reduced at 6 h in GA-MSCs treated with the virus compared with control (Fig. 4A). The results were also quantified; the tube segment lengths (*P* < 0.01, 53.56 ± 4.023 vs. 17.83 ± 4.426) and number of tubes (*P* < 0.01, 57.5 ± 4.95 vs. 13.5 ± 2.121) of the GA-MSCs treated with Ad5-Ki67/IL-15 were significantly shorter than those of control (Fig. 4B, C). Furthermore, GA-MSCs incubated with supernatants from Ad5-Ki67/IL15-treated U251 cells had significantly decreased angiogenic capacity at 6 h compared with those incubated with conditioned medium from U251 cells; the tube segment lengths (*P* < 0.05, 41.91 ± 2.291 vs. 24.55 ± 1.782) and number of tubes (*P* < 0.05, 45.5 ± 6.364 vs. 25 ± 1.414) of the GA-MSCs treated with Ad5-Ki67/IL-15-CM were significantly shorter than those of control (Fig. 4D–F). This provides a new strategy that inhibited tumor angiogenesis to treat GBM using Ad5-Ki67/IL-15 targeting GA-MSCs in future.

### Ad5-Ki67/IL-15 overcame GA-MSCs to enhance therapeutic efficacy and reduce PD-L1 expression in glioma cells

**Fig. 5:**
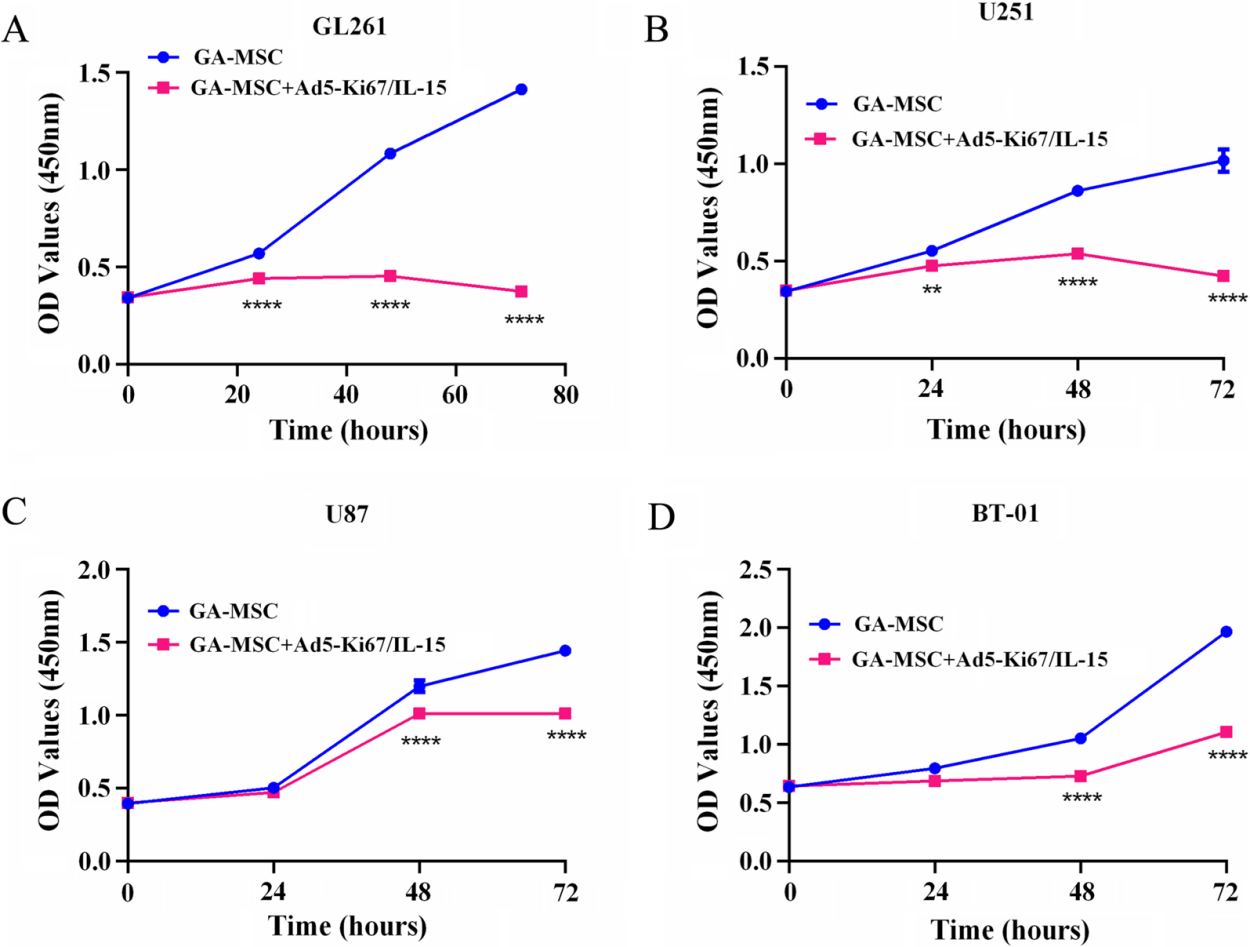
Ad5-Ki67/IL-15 could overcome GA-MSCs to effectively kill glioma cells. **A** The effects of GA-MSCs treated with the virus on mouse glioma cell GL261 were determined by CCK-8. The virus-treated GA-MSCs significantly inhibited the proliferation of glioma cells compared to than that of GA-MSCs. **B** Human glioma cell U251. **C** Human glioma cell U87. **D** Human glioma cell BT-01. All data are presented as mean ± SD of three independent experiments. *P < 0.05, **P < 0.01, ***P < 0.001, ****P < 0.0001

**Fig. 6:**
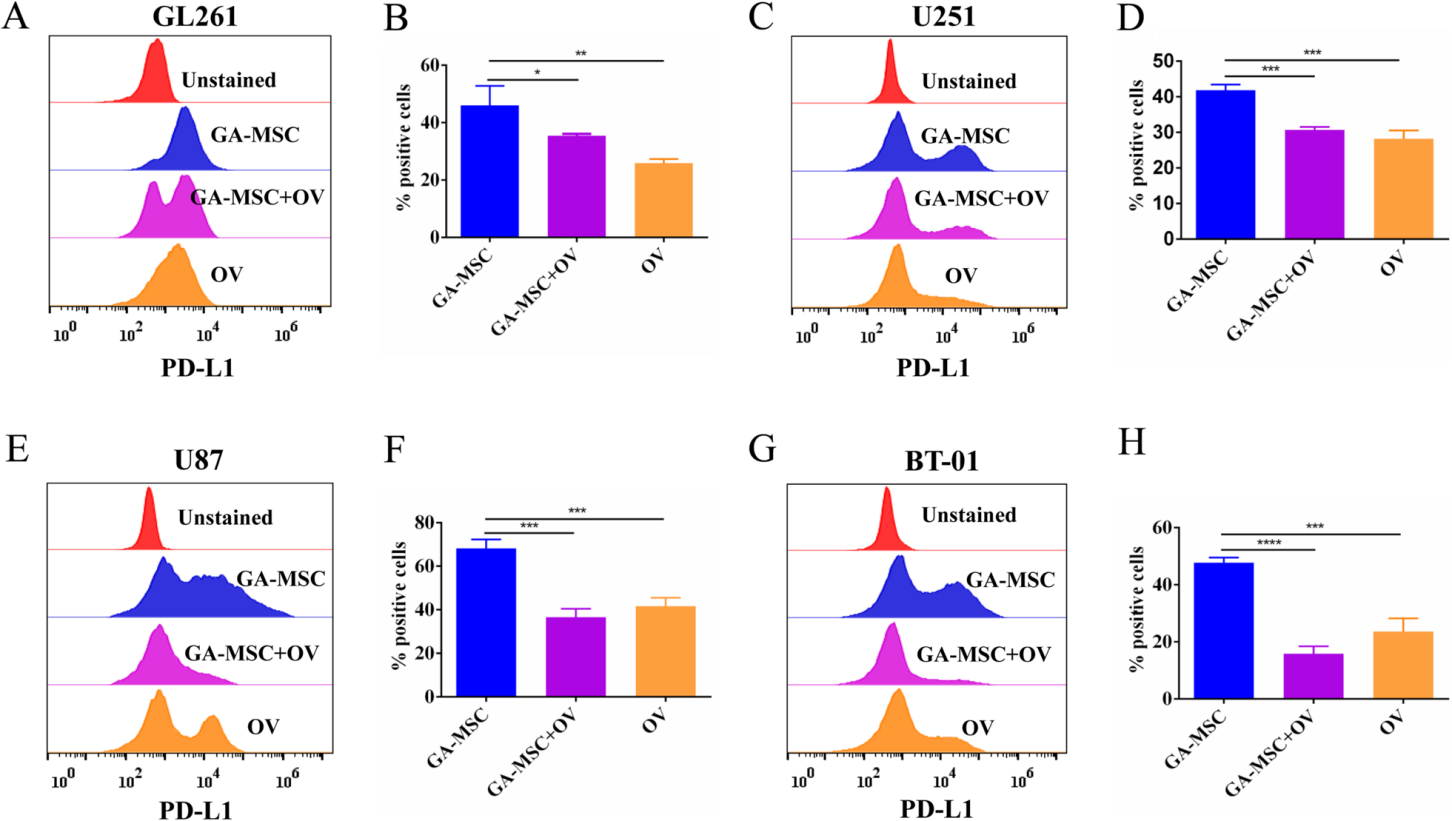
GA-MSCs-mediated PD-L1 expression was attenuated by Ad5-Ki67/IL-15 in vitro. **A** and **B** Glioma cell GL261 cocultured with GA-MSCs with or without the virus for 72 h. Flow cytometry revealed that the virus could decrease PD-L1 expression in glioma cells despite the presence of GA-MSCs. **C** and **D** Human glioma cell 251. **E** and **F** Human glioma cell U87. **G** and **H** Human glioma cell BT-01. All data are presented as mean ± SD of three independent experiments. *P < 0.05, **P < 0.01, ***P < 0.001, ****P < 0.0001

Prior studies demonstrated that GA-MSCs promoted glioma growth and progression [8, 10]. To evaluate the effects of Ad5-Ki67/IL-15 on PD-L1 expression in glioma cells, different glioma cells GL261, U251, U87 and BT-01, respectively, cocultured with GA-MSCs with the virus for 24 h, 48 h and 72 h, we confirmed that the virus has the potential to overcome GA-MSCs to effectively kill glioma cells (Fig. 5A–D). In addition, we found Ad5-Ki67/IL-15 could attenuate PD-L1 expression of mouse glioma cells induced by GA-MSCs in GBM treatment (Fig. 6A, B, *P* < 0.05, 46.03 ± 6.793 vs. 35.37 ± 0.666). Moreover, the virus also decreased GA-MSC-mediated PD-L1 in human glioma cells U251 (*P* < 0.001, 41.83 ± 1.582 vs. 30.63 ± 0.834), U87 (*P* < 0.001, 68.17 ± 4.055 vs. 36.6 ± 3.736) and BT-01 (*P* < 0.001, 47.67 ± 1.818 vs. 15.83 ± 2.579) (Fig. 6C–H). In addition, we also revealed that PD-1 in mouse lymphocytes was increased compared with controls and the virus could reduce GA-MSC-induced PD-1 expression and T cell inhibition (Additional file 2: Fig. S1 and Additional file 3: Fig. S2). Therefore, although GA-MSCs promote PD-L1 upregulation in glioma, the virus can attenuate this property of GA-MSCs.

### Ad5-Ki67/IL-15 induced antitumor efficacy despite the presence of GA-MSCs in GBM model

**Fig. 7:**
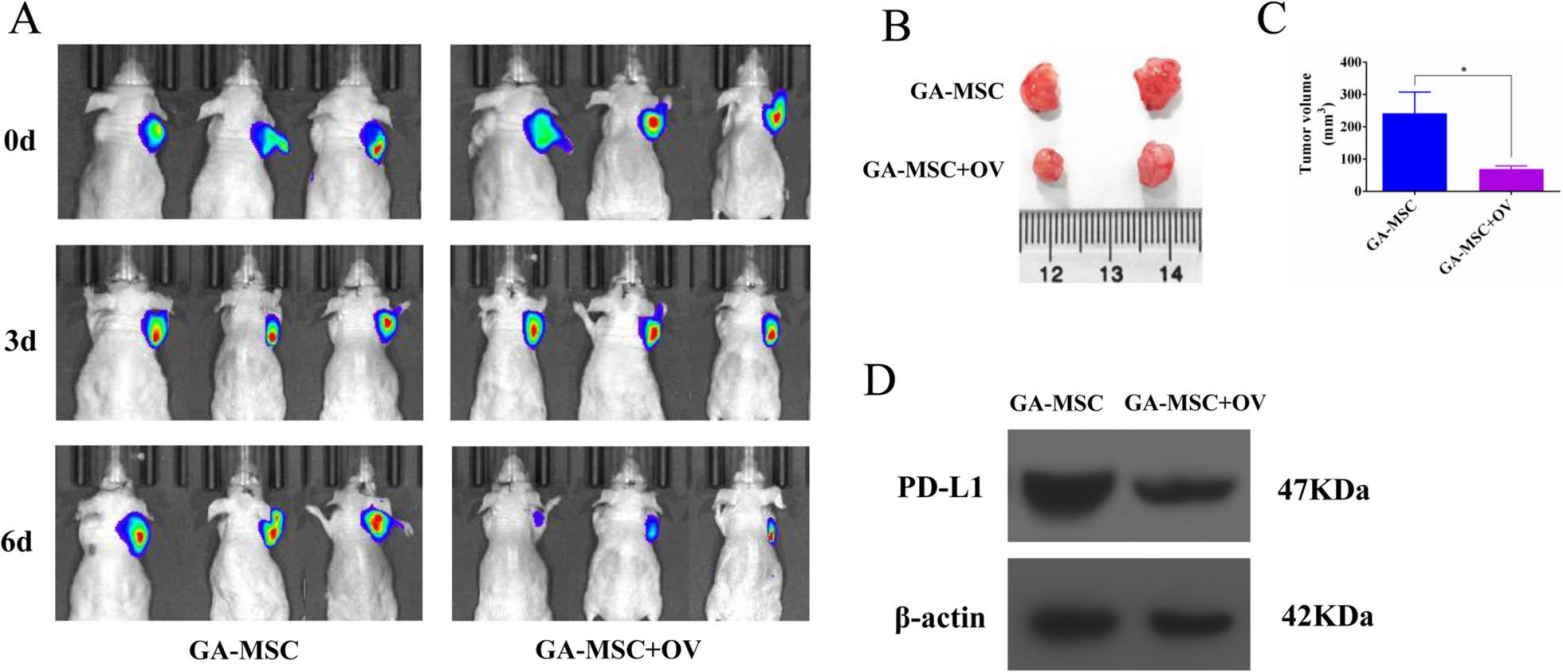
Ad5-Ki67/IL-15 induced potent antitumor efficacy in GBM model with GA-MSCs. **A** BALB/nu-c mice were subcutaneously injected with 100 μl PBS suspension containing 1 × 10^6^ U87-Luc cells and divided into groups according to tumor growth conditions based on live-animal IVIS imaging 14 days post tumor cell injection. Subsequently, the 100 μl GA-MSCs suspension (1 × 10^5^ cells) or Ad5-Ki67/IL-15 (3 × 10^11^ vp) plus GA-MSCs (1 × 10^5^ cells) were injected into the tumor sites. IVIS imaging was also performed 3 and 6 days post virus injection. **B** and **C** Representative mice from xenograft experiments in which U87 cells with GA-MSCs (upper) or U87 cells with GA-MSCs plus OV (lower) were injected into the right underarm of nude mice. Obviously, the sizes of the GA-MSCs plus OV group tumors were lower than those of their GA-MSCs counterparts. *P < 0.05. **D** The tumor specimens were acquired to evaluate PD-L1 expression. The images of blots were cropped from different gels; moreover, exposure time of β-actin and PD-L1 was, respectively, 15 s and 45 s. Western blotting confirmed PD-L1 in GA-MSC plus virus was reduced compared to the GA-MSCs group

To investigate the therapeutic efficacy of the virus in vivo, we established a human GBM model. IVIS images of mice bearing U87-derived tumors showed that tumor size was significantly decreased in the Ad5-Ki67/IL-15 plus GA-MSCs group compared with the GA-MSCs alone, especially 6 days after the virus injection (Fig. 7A). Intracranial tumors of glioma-bearing mice were collected; we found significant reductions in tumor volumes after the addition of Ad5-Ki67/IL-15 treatment (Fig. 7B, C). Moreover, western blotting revealed that PD-L1 expression in tumor was downregulated by Ad5-Ki67/IL-15 (Fig. 7D). These results suggested that oncolytic Ad5-Ki67/IL-15 can induce significant antitumor even if the presence of GA-MSCs in glioma.

## Discussion

GBM is highly immunosuppressive and vascular tumor, resulting in poor immunotherapy outcomes. In this study, we for the first time discover that GA-MSCs contribute to the formation of PD-L1 upregulation in glioma. Further studies reveal that oncolytic Ad5-Ki67/IL-15 effectively reduces neovascularization in GA-MSCs, indicating potential anti-tumor angiogenic capacity. Furthermore, the virus decreased PD-L1 expression of glioma and induced antitumor efficacy by targeting GA-MSCs. To our knowledge, this is the first description of GA-MSC-mediated PD-L1 expression in brain glioma.

GA-MSCs, as resident stromal components in the unique microenvironment of human brain tumors, severely affect prognosis and survival time of GBM patients [9]. The fraction of MSCs in glioma tissues may be distinct in different patients, even within the same histopathological characteristics [9]. This signifies that therapeutic strategy targeting MSCs may vary from patient to patient. Therefore, deeper research about GA-MSCs in glioma will provide a reliable perspective for the development of individualized treatment in future. Previous studies about GA-MSCs mainly focused on the effects of tumor initiation, progression, driving GSCs proliferation and therapeutic resistance [8, 26, 27]. However, there are few studies on its immune regulation in glioma. An important finding of this study was that GA-MSCs can upregulate PD-L1 expression and can be downregulated by the virus. Moreover, the virus was still able to achieve the desired antitumor effect in a human GBM model containing GA-MSCs.

The source of GA-MSCs is unknown, and the most probable explanation for their presence is that the tumor recruits them from normal tissues [26, 28], as well as GSCs differentiation [26]. The involvement of MSCs in angiogenic activity has been well established [10, 29]. Furthermore, GA-MSCs have the potential to differentiate into pericytes and maintain tumor vascular structure, and the GA-MSCs with CD90 low expression show a more active angiogenic capacity, which contribute to glioma progression [10, 30]. Studies on GA-MSCs have mainly focused on their effects on tumor progression [8, 26], whereas their immunological properties and treatments targeting GA-MSCs have not been reported. Our study found that GA-MSCs showed potent immunosuppressive characteristics, the increased numbers of both PD-1^+^ and PD-L1^+^cells were found.

Recently, the effects of MSCs on T cells have been intensively studied. Immunosuppressive factors released by MSCs, such as TGF-β, IL-10 and HGF, which are thought to be main causes of the inhibition of T cell proliferation [14]. These factors lead to cyclin D2 downregulation and p27kip1 upregulation in T cells, thereby arresting proliferation in the G1 phase, and have been shown to induce immunosuppression within tumors [15, 18, 19]. MSCs also generate a variety of chemokines and adhesion molecules, which play important parts in lymphocyte recruitment, thus ensuring their immunosuppressive function. Increased expression of immunoregulatory and adhesion molecules is essential for effective T cell inhibition [17, 31, 32]. In addition, MSCs have the capacity to alter the activation and differentiation of T cells. There is evidence that MSCs decrease IFN-γ and IL-17 secretion by T cells but promote IL-10 production by antagonizing the differentiation of Th1 and Th17 cells, thereby inducing the generation of regulatory T cells [11, 13, 33]. Furthermore, our study demonstrated that GA-MSCs can upregulate PD-L1 expression in glioma, indicating a potential immunosuppressive property.

PD-L1 is expressed in various tissues and is often upregulated in malignant tumors; higher PD-L1 expression in GBM has been found to be correlated with poorer patient prognosis in some studies [34, 35]. PD-L1 is an immune inhibitory receptor ligand that leads to immune cell dysfunction and apoptosis by binding to its receptor PD-1 [36]. This facilitates the immunosuppressive microenvironment and tumor progression. An increasing number of studies hint that PD-1/PD-L1 is a promising target to reverse the immune evasion of GBM [37–39]. In this study, we demonstrate that GA-MSCs have the potential to increase PD-L1 of glioma and PD-1 in lymphocytes, indicating a potential immunosuppressive capacity. PD-L1 hinder T cell activation, especially during the effector phase, leads to T cell exhaustion during chronic antigen exposure, and inhibits NK cells and B cells; however, antagonists of PD-L1 or its ligands have been shown to reverse immune function at least in part and to enhance OV therapy [40, 41]. Belcaid et al. found that Delta24-RGD oncolytic virus therapy overcome glioma-induced immune suppression and increase PD-1^+^ tumor-infiltrating CD8^+^ T cells [42]. Our investigation revealed that oncolytic Ad5-Ki67/IL-15 downregulate PD-L1 expression in glioma with the presence of GA-MSCs.

There are some limitations in our study. For example, the GA-MSCs that we used in the experiment were derived from patients diagnosed with glioma, but the fraction of the cells is not abundant in some glioma tissues, and that the heterogeneity of MSC will present great challenges for this research. Our data demonstrate that OVs improve GBM treatment by remodeling GA-MSCs-mediated PD-L1 expression and angiogenesis. However, we do not determine the interaction of GA-MSCs-mediated PD-L1 and immune cells and also not evaluated the specific manner by which OVs affect angiogenic capacity in this study.

## Conclusions

In summary, our results confirmed that GA-MSCs participate in the upregulation of PD-L1 expression of glioma. Our findings further indicate that GA-MSC-mediated PD-L1 can be attenuated by Ad5-Ki67/IL-15 in treatment, thereby enhancing the effectiveness of immunotherapy in GBM. This study indicates a potential new approach for GBM treatment using Ad5-Ki67/IL-15 to target not only tumor cells but also MSCs.

## Supplementary Information

**Additional file 1:** Schematic diagram of study about OVs and GA-MSCs.

**Additional file 2: Fig. S1.** Ad5-Ki67/IL-15 reduced GA-MSCs-mediated PD-1 expression in lymphocytes. Mouse lymphocytes cocultured with GA-MSCs with or without Ad5-Ki67/IL-15 for 72 h. Flow cytometry showed that GA-MSCs promoted PD-1 upregulation and the virus could decrease PD-1 expression induced by GA-MSCs. All data are presented as mean ± SD of three independent experiments. *P < 0.05, **P < 0.01, ***P < 0.001, ****P < 0.0001.

**Additional file 3: Fig. S2.** Ad5-Ki67/IL-15 improved GA-MSCs-mediated T cell inhibition. Mouse lymphocytes cocultured with GA-MSCs with or without Ad5-Ki67/IL-15 for 72 h. Flow cytometry showed that the virus could attenuate GA-MSCs-mediated T cell inhibition induced by GA-MSCs.

## Data Availability

All data analyzed during this study are included in this published article.

## Abbreviations

TME: Tumor microenvironment
GA-MSCs: Glioma-associated mesenchymal stem cells
MSCs: Mesenchymal stem cells
BM-MSCs: Bone marrow mesenchymal stem cells
GSCs: Glioma stem cells
PD-1: Programmed death protein 1
PD-L1: Programmed death ligand 1
PGE2: Prostaglandin E2
TGFβ: Transforming growth factor beta
IL-10: Interleukin-10
IDO: Indoleamine 2,3-dioxygenase
MOI: Multiplicity of infection
OAd: Oncolytic adenovirus
OV: Oncolytic virus
