# Supplementary figures and images for: Glioma-associated mesenchymal stem cells-mediated PD-L1 expression is attenuated by Ad5-Ki67/IL-15 in GBM treatment
Source: Stem Cell Res Ther. 2022 Jun 28;13:284. doi: 10.1186/s13287-022-02968-z (PMC9241198; doi:10.1186/s13287-022-02968-z)

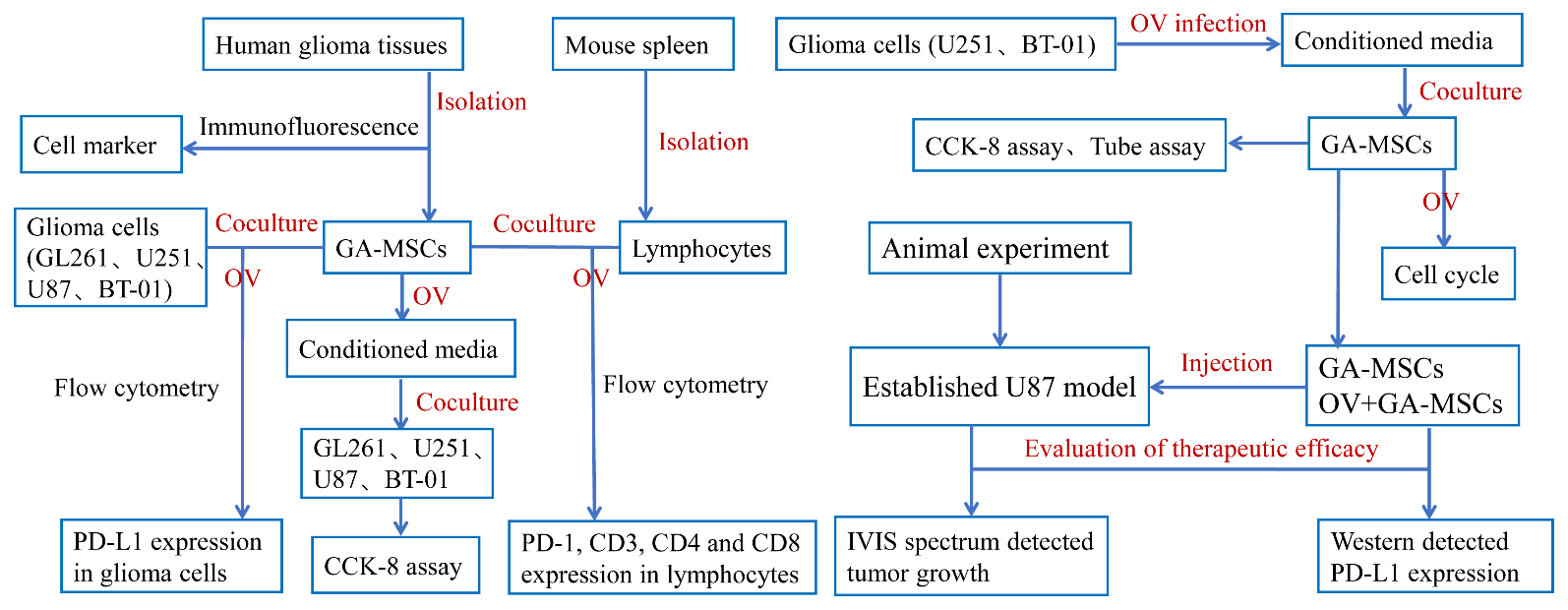

Supplement: Supplementary file 1 — Additional file 1: Schematic diagram of study about OVs and GA-MSCs. [file 13287_2022_2968_MOESM1_ESM.docx]

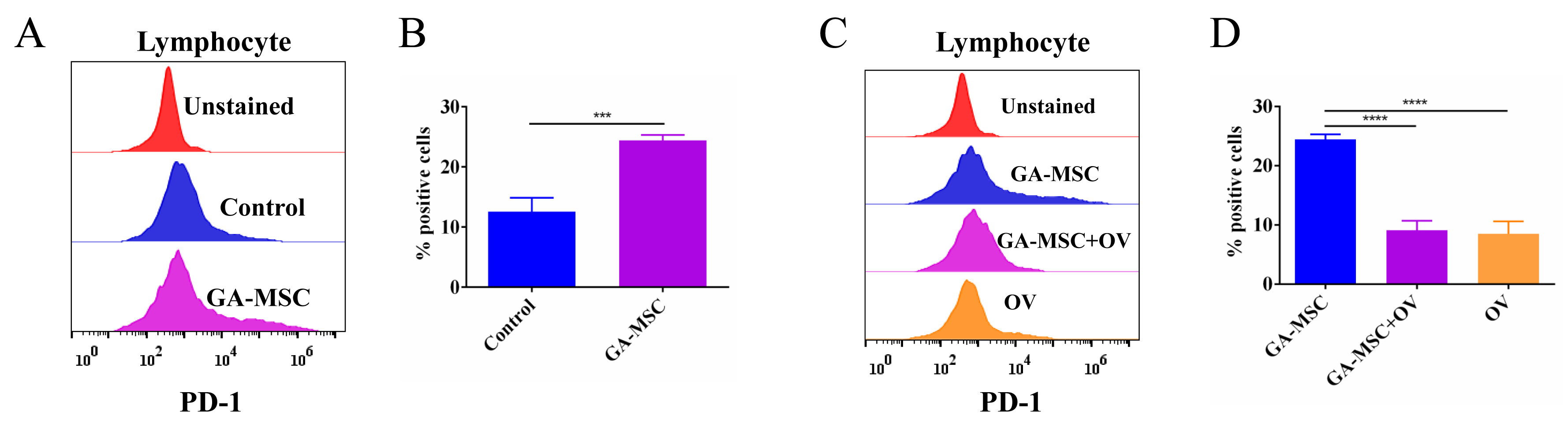

Supplement: Supplementary file 2 — Additional file 2: Fig. S1. Ad5-Ki67/IL-15 reduced GA-MSCs-mediated PD-1 expression in lymphocytes. Mouse lymphocytes cocultured with GA-MSCs with or without Ad5-Ki67/IL-15 for 72 h. Flow cytometry showed that GA-MSCs promoted PD-1 upregulation and the virus could decrease PD-1 expression induced by GA-MSCs. All data are presented as mean ± SD of three independent experiments. *P < 0.05, **P < 0.01, ***P < 0.001, ****P < 0.0001. [file 13287_2022_2968_MOESM2_ESM.tif]

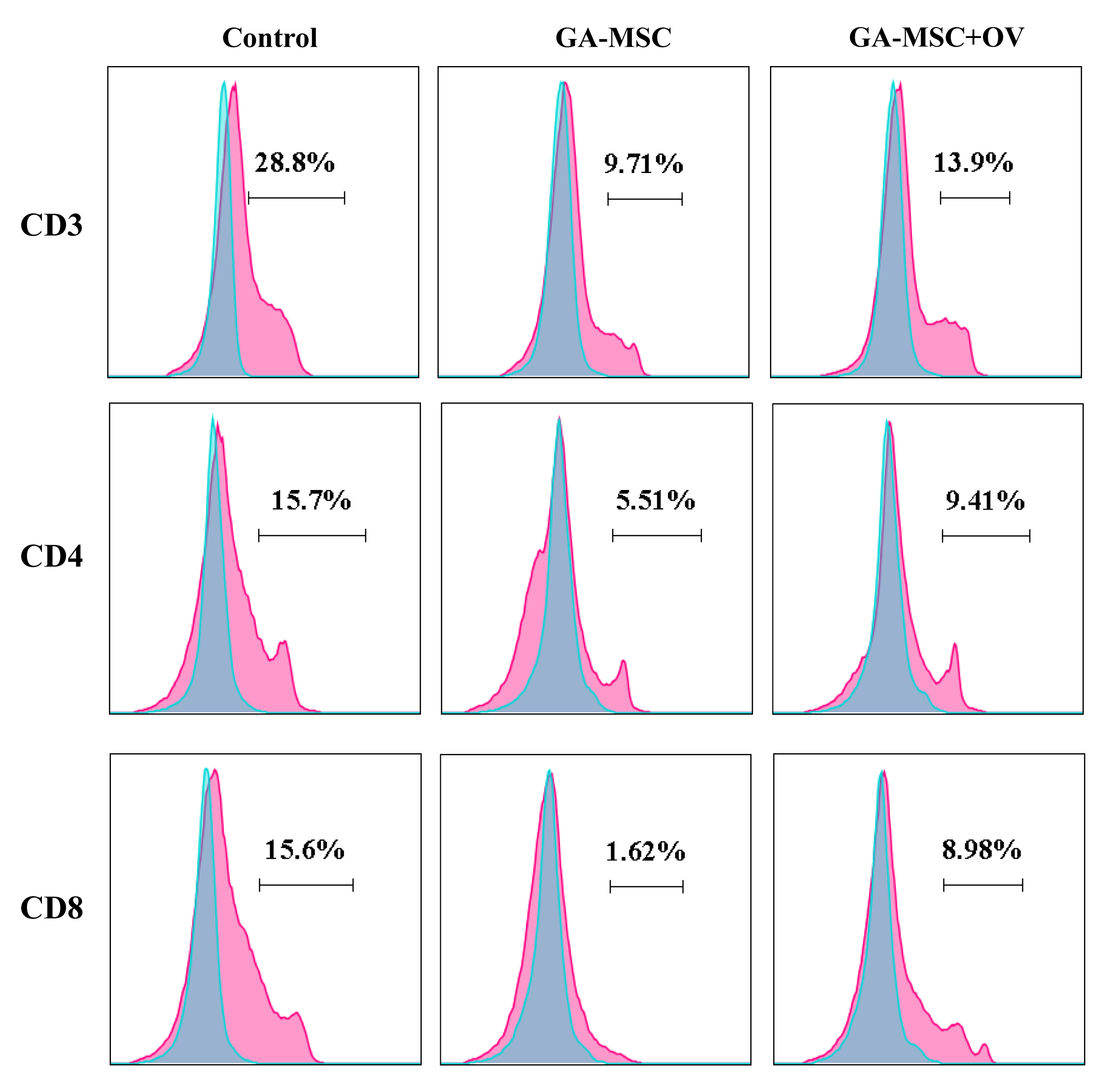

Supplement: Supplementary file 3 — Additional file 3: Fig. S2. Ad5-Ki67/IL-15 improved GA-MSCs-mediated T cell inhibition. Mouse lymphocytes cocultured with GA-MSCs with or without Ad5-Ki67/IL-15 for 72 h. Flow cytometry showed that the virus could attenuate GA-MSCs-mediated T cell inhibition induced by GA-MSCs. [file 13287_2022_2968_MOESM3_ESM.tif]
